# Supplementary material for: Small-Molecule-Mediated Suppression of BMP Signaling by Selective Inhibition of BMP1-Dependent Chordin Cleavage
Source: Int J Mol Sci. 2023 Feb 21;24(5):4313. doi: 10.3390/ijms24054313 (PMC10001940; doi:10.3390/ijms24054313)
Supplement: Supplementary file 1 [file ijms-24-04313-s001.zip › ijms-2202932-supplementary.pdf]

Supplementary figures, table, and legends

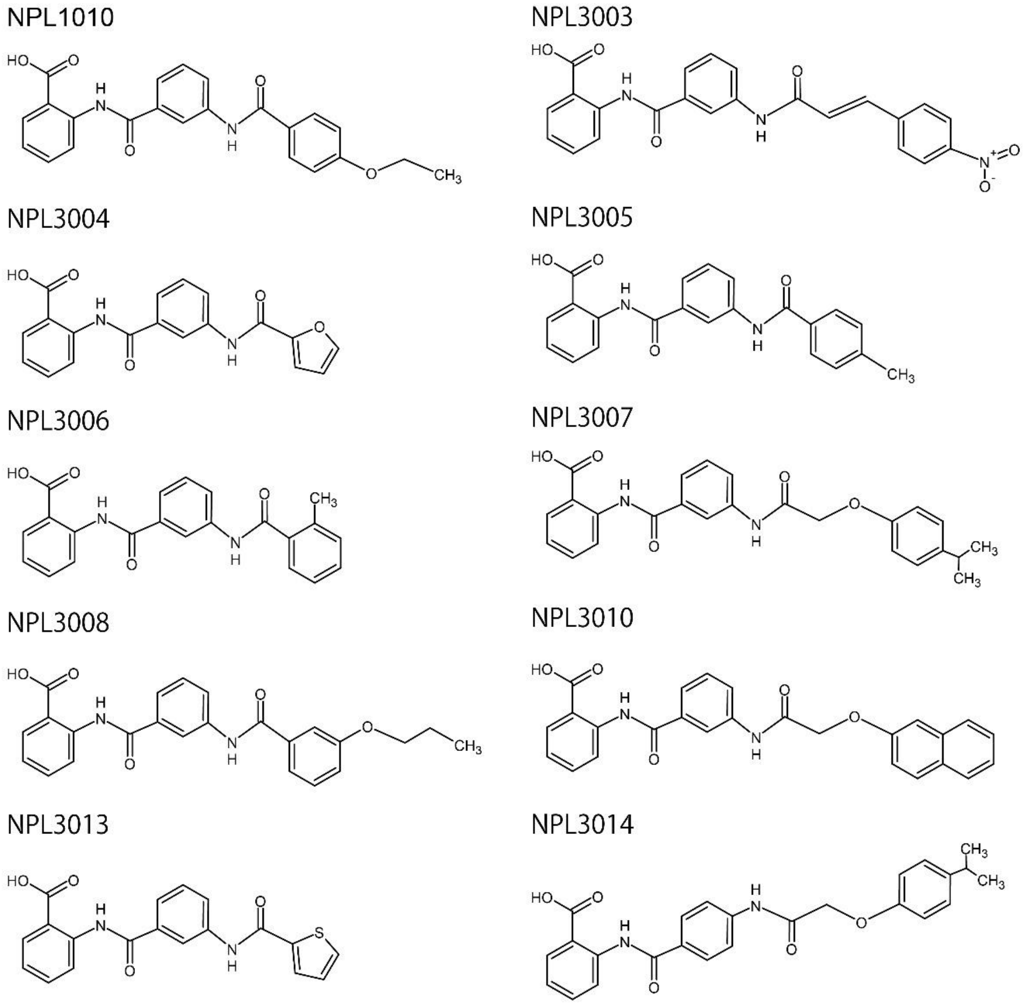

Figure S1: Structural formulas of candidate compounds.

The structural formulas of ten candidate compounds targeting the PDZ domain that were screened *in silico*.

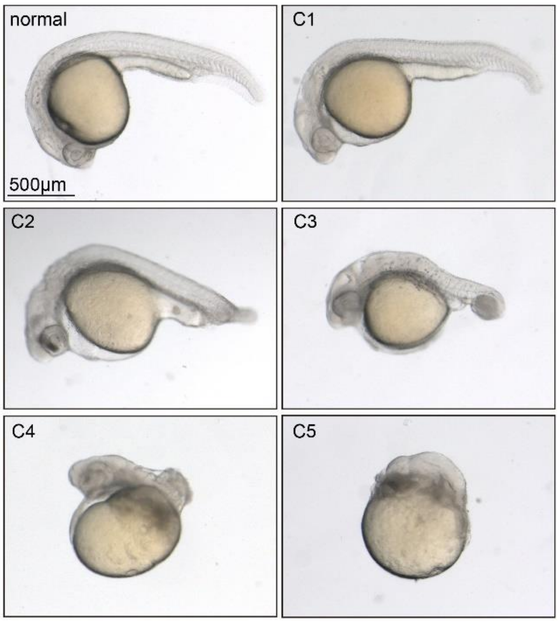

Figure S2: Representative images of C1–C5 categories.

Representative normal and dorsalized phenotypes of C1–C5 categories are shown in the photographs. The phenotypes are more severe in the order of C1 to C5. Scale bar, 500 μm.

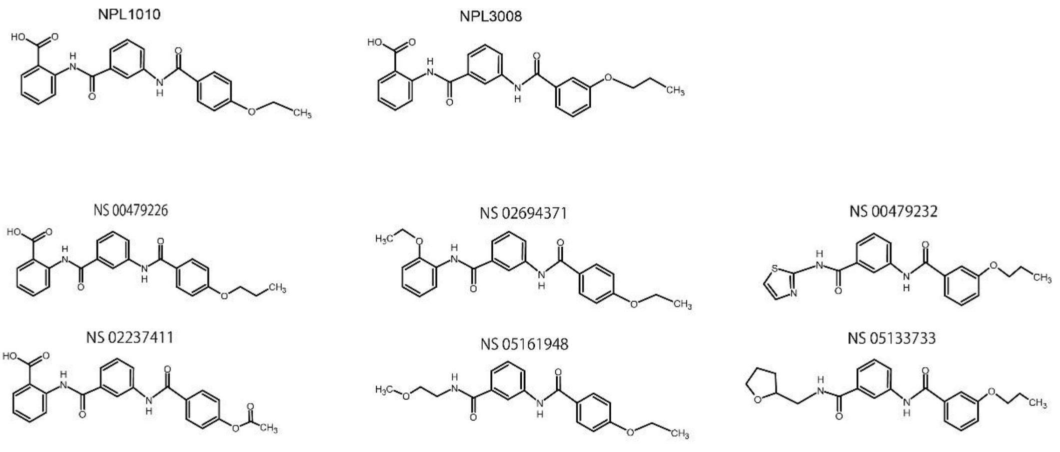

Figure S3: Structural formulas of additional candidate compounds.
The structural formulas of candidate compounds that are derivatives of NPL1010 or NPL3008.

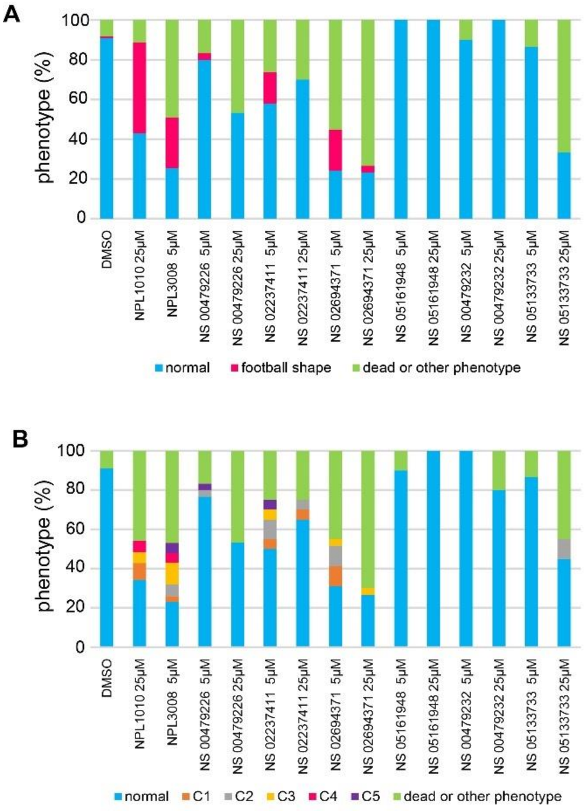

Figure S4: Proportion of zebrafish displaying phenotypes after treatment with the NPL1010 and NPL3008 analogs.

(A) Proportion of zebrafish displaying phenotypes after treatment with NPL1010 and NPL3008 analogs at 11 hpf. *n* = 10-125. (B) Proportion of zebrafish displaying phenotypes after treatment with NPL1010 and NPL3008 analogs at 24 hpf. *n* = 10-125.

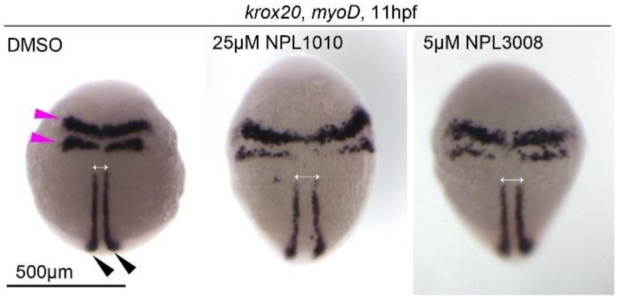

Figure S5: NPL1010 and NPL3008 induce dorsalization.
In situ hybridization against *myoD* and *krox20*. NPL1010- and NPL3008-treated embryos showed an expanded dorsal structure at 11 hpf. Representative normal, NPL1010-treated, and NPL3008-treated phenotypes are shown in the photographs. Dorsal view, anterior to the top. The magenta arrowheads indicate the *krox20* expression domain. The black arrowheads indicate the *myoD* expression domain. Scale bar, 500 μm. The double-headed arrows indicate the width of the *myoD* expression domain.

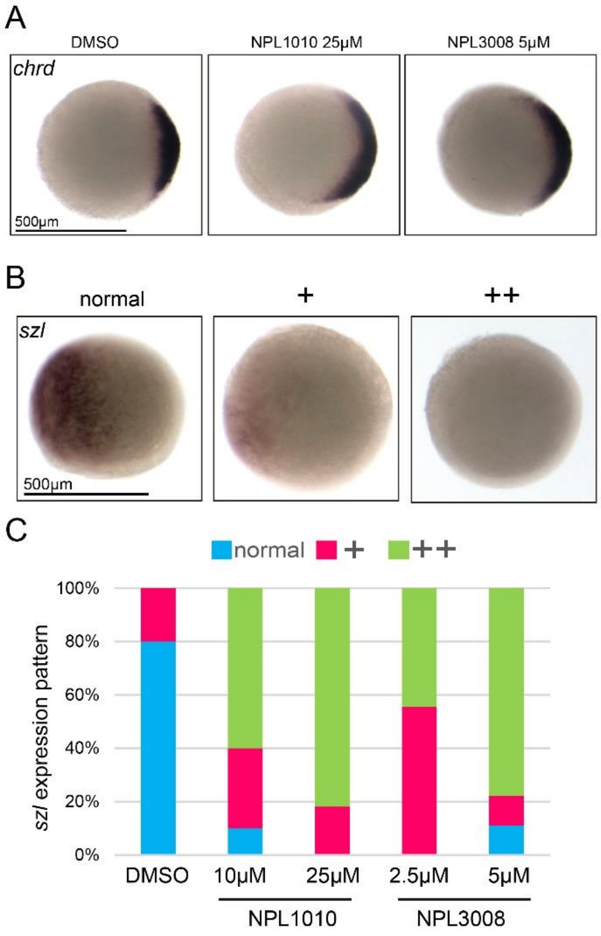

Figure S6: Supporting data for Figure 2.

(A) Representative expression pattern of *chrd* in normal, NPL1010-treated, and NPL3008-treated embryos shown in the photographs. (B) The classification of the *szl* expression pattern, normal, slight reduction/mild phenotype (+), and strong reduction/severe phenotype (++) at 6 hpf with representative expression patterns shown in the photographs. Animal pole view, ventral to the left. Scale bar, 500 μm. (C) Proportion of NPL1010- or NPL3008-treated embryos displaying *szl* expression patterns. *n* = 9-11.

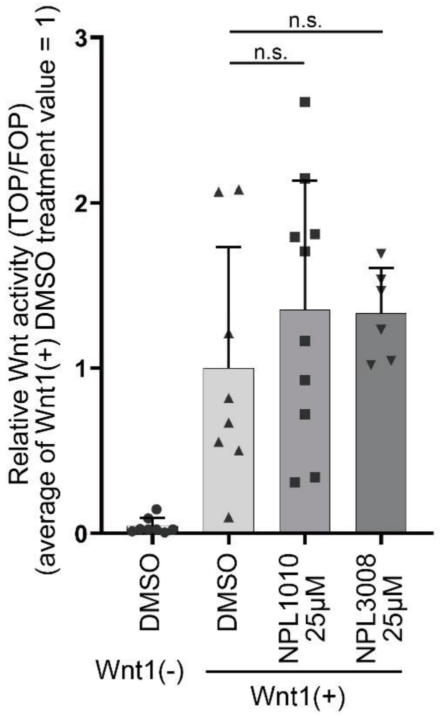

Figure S7: NPL1010 and NPL3008 do not affect Wnt/β-catenin signaling.
Relative Wnt activity (TOP/FOP) is shown. The average of Wnt1(+) DMSO treated sample values equal 1. NPL1010 and NPL3008 did not reduce the Wnt activity. n.s., no significant difference (Dunnett's test). The error bar indicates +s.d. with individual data shown. n = 6-10.

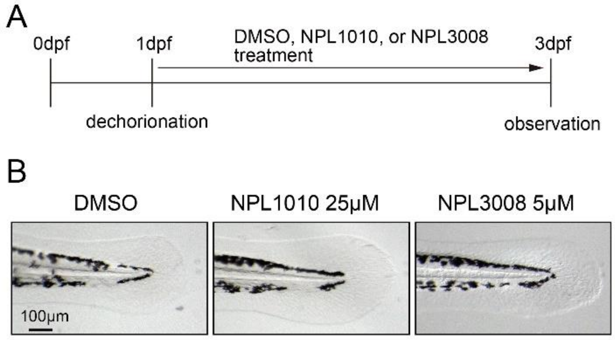

Figure S8: The ruffled fin phenotype is not induced NPL1010 or NPL3008 treatment.

(A) Schematic diagram of the analysis of the effects of NPL1010 and NPL3008 on fin morphogenesis. (B) The ruffled fin phenotype, which is observed in *bmp1a* mutant and morphant, was not induced in NPL1010 or NPL3008 treated embryos. Representative DMSO, NPL1010, and NPL3008 treated embryo fin are shown in the photographs. Scale bar, 100 μm.

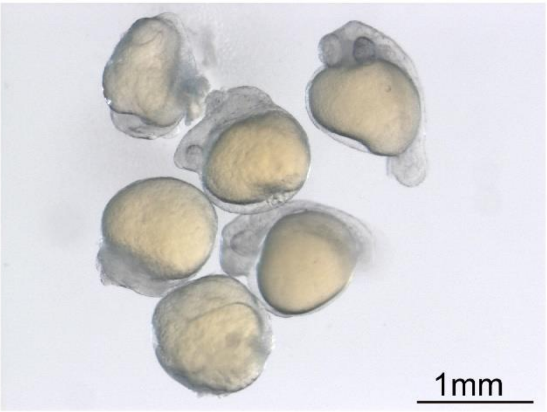

Figure S9: Chrd-Myc can induce the dorsalized phenotype.
Representative phenotype of 500pg *chrd* mRNA injected 24hpf embryos are shown. The injected embryos showed severe dorsalized phenotype. Scale bar, 1mm.

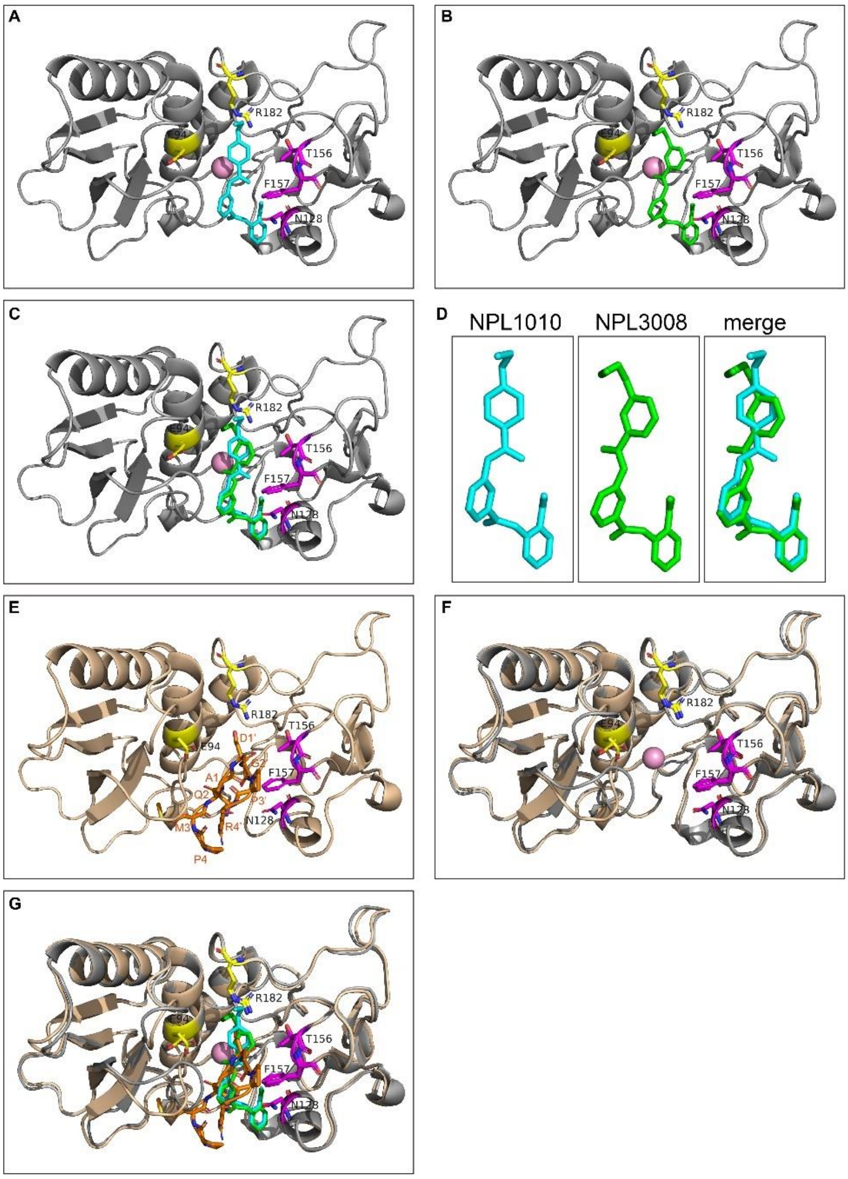

Figure S10: The docking simulation of NPLs and the BMP1 catalytic domain.
(A) The docking simulation of NPL1010 and the BMP1 catalytic domain. (B) The docking simulation of NPL3008 and the BMP1 catalytic domain. (C) Merged view of (A) and (B). (D) Enlarged view of the NPL1010 (blue) and NPL3008 (green) docking poses in (A)-(C). (E) The simulated interaction of the Chrd C-terminal region and BMP1 catalytic domain. The cleavage site of the Chrd C-terminus (P4-R4’) is visualized in orange. (F) Structure alignment of the BMP1 catalytic domain in docking simulation (A-C, gray) and AlphaFold simulation (E, wheat color). (G) Merged view of the images shown in (C) and (E). Pink, zinc ion; magenta, Asn128/Thr156/Phe157; yellow, Glu94/Arg182.

#	Supplier	catalog number
NS 00479226	Vitas-M	STK156287
NS 02237411	Vitas-M	STK299714
NS 02694371	SPECS	AN-652/43163216
NS 05161948	Vitas-M	STL068608
NS 00479232	Vitas-M	STK051079
NS 05133733	Vitas-M	STL068169

Table S1. Supplier and catalog numbers for the chemical compounds used in the structure-activity relationship experiment.
